# Investigating Endoparasites in Captive Birds of Prey in Italy

**DOI:** 10.3390/ani14243579

**Published:** 2024-12-11

**Authors:** Carolina Allievi, Sergio A. Zanzani, Fulvio Bottura, Maria Teresa Manfredi

**Affiliations:** 1 Department of Veterinary Medicine and Animal Sciences, Università Degli Studi di Milano, Via Dell’ Università 6, 26900 Lodi, Italy; sergio.zanzani@unimi.it (S.A.Z.); mariateresa.manfredi@unimi.it (M.T.M.); 2 Research Laboratory of Animal Parasitic Diseases and Zoonoses (ParVetLab), Università Degli Studi di Milano, Via Dell’ Università 6, 26900 Lodi, Italy; 3 Independent Researcher, 22100 Como, Italy; fulviobottura@gmail.com

**Keywords:** birds of prey, captivity, endoparasites, haemoparasites, FLOTAC^®^, Italy

## Abstract

Birds of prey can be parasitised by several endoparasites that can coexist without clinical signs of disease or occur in conjunction with stressful events. Because the number of birds of prey kept in captivity is copiously increasing due to their use for bird control, breeding programs, exhibition and falconry, the main endoparasites of 81 apparently healthy captive birds of prey from northern Italy were investigated by examining faecal and blood samples. Faeces were analysed by a quali-quantitative technique, i.e., the FLOTAC^®^ basic technique, employing potassium iodomercurate flotation solution, while blood smears were stained to detect haemoparasites. Risk factors were further assessed. Considering gastrointestinal parasites, an overall prevalence of 41.7% was recorded, and 50% of Accipitriformes, 43% of Falconiformes and 33.3% of Strigiformes tested positive for at least one parasite taxon. Moreover, age and diet were associated with an increased risk of infection. As for haemoparasites, a prevalence of 18.2% was evidenced, and none of the risk factors were associated with prevalence. The results of this study highlighted the importance of monitoring the endoparasites of captive birds of prey with a highly sensitive copromicroscopic technique to target medical treatments, improve housing conditions and conduct epidemiological studies aimed at wildlife conservation and management.

## Simple Summary

Although there are many birds of prey kept in captivity, studies on parasitoses that can affect them are scarce and fragmentary, especially in the Italian framework. Therefore, the purpose of this study was to investigate the main endoparasites of several captive birds of prey housed in different facilities in northern Italy by performing both faecal analysis, with a copromicroscopic method characterised by high sensitivity, and blood analysis for the detection of haemoparasites. The present study demonstrated a high parasite burden, underscoring the importance of performing parasitological screening tests on these animals to ensure their welfare and conservation.

## 1. Introduction

Birds of prey can be kept in captivity either permanently (i.e., in wildlife parks or breeding programs or for exhibition or falconry) or temporarily (i.e., for rehabilitation in rescue centres). Their proper training to live in captivity is complex and can be risky for animal health and welfare as it can lead to the development of aggressiveness, wrong imprinting and stress [1].

As predatory animals positioned at the top of the food chain, birds of prey could be used as sentinels for many circulating pathogens affecting birds, pets, livestock and humans [2,3]. Indeed, captive and free-ranging birds of prey can be affected by a wide range of endoparasites, including nematodes, trematodes, cestodes, protozoans and acanthocephalans [4].

In most scenarios, parasites in birds of prey can coexist without apparently causing any harm or deleterious effects on the host. Nevertheless, under certain circumstances, such as stressful events, they can trigger anorexia, diarrhoea, apathy and even death [5,6]. These symptoms are more common in captive animals, as the high density and limited space available for each animal could determine an increase in infection rates and clinical forms [6,7,8]. Particularly, some protozoan and helminth species can affect both flight and predatory ability and predispose them to secondary trauma; in addition, injuries caused by endoparasites could be compounded by secondary bacterial infections [8,9].

The main protozoa affecting birds of prey, such as *Caryospora* spp., *Cryptosporidium* spp., *Eimeria* spp., *Sarcocystis* spp. and *Toxoplasma gondii*, are often detected in young animals or those with compromised immune systems [5,10,11,12,13]. Among haemoprotozoa, some of the most significant are *Haemoproteus* spp., *Leucocytozoon* spp. and *Plasmodium* spp., which are transmitted to birds through insect bites [5,14,15,16].

Regarding helminth infections, nematodes represent the largest and most pathogenic group with several families, followed by trematodes, particularly of the class Digenea, and cestodes [8,17,18,19]. Data on their prevalence and abundance would be of great value in defining their impact on bird health and conservation and in establishing appropriate control measures to reduce the spread of infectious diseases [8,20].

In Italy, studies conducted on birds of prey are limited, showing different prevalence values depending on the type and number of birds sampled: in southern Italy, a parasite prevalence of 35.6% in zoo and pet birds was shown, while in central Italy, a positivity of 66.7% to nematodes and protozoa was evidenced in a small population (*n* = 29) of birds housed in wildlife recovery centres [7,8]. It is important to emphasise that in both of the above-mentioned studies, a qualitative copromicroscopic method was employed, i.e., the faecal flotation test, which does not allow for an estimation of the parasite load and has a limited sensitivity in the case of low excretion of parasitic elements [21].

Because captive breeding of birds of prey is on the rise and parasitological studies conducted on them are almost absent in Italy, the present study examined faecal and blood samples from several birds kept in captivity with the aim of determining the parasite prevalence, reporting their abundances for the first time employing a highly sensitive copromicroscopic quantitative technique, and assessing the main risk factors.

## 2. Materials and Methods

### 2.1. Study Population and Sample Collection

The survey was conducted from July 2018 to January 2019 and included 81 birds of prey: 30 belonging to the order Accipitriformes, 17 to Falconiformes and 34 to Strigiformes (Table 1). All animals were in good physical condition and had no symptoms attributable to the presence of parasites. Samples were collected in four centres located in northern Italy, between the Lombardy (3) and Piedmont (1) regions, comprising an educational farm, a wildlife recovery centre, a breeding farm and a zoological garden.

All species involved in the study are listed on the Red List of Threatened Species of the International Union for the Conservation of Nature (IUCN), the world’s largest database of information on the conservation status of animal and plant species, in which animals are divided into nine levels according to a precise category of threat [22] “https://www.iucnredlist.org (accessed on 3 April 2024)”.

At the same time as sampling, an individual form was filled out collecting information about the animals: age, diet, bird family of belonging, interaction with other animals and housing arrangements.

### 2.2. Laboratory Analysis

Faeces were collected from 72 of the 81 sampled animals, immediately after the cloacal expulsion. Samples were then placed in a container suitable for storage and kept at a temperature of +4 °C until laboratory analysis, which was performed within two days after sampling.

Faeces were analysed with the FLOTAC^®^ basic technique (University of Naples Federico II, Naples, Italy) using potassium iodomercurate flotation solution, FS8 (HgI_4_K_2_; specific gravity, s.g. = 1440), which allows for both qualitative and quantitative detection of parasitic elements with an analytic sensitivity of one egg/oocyst/larva per gram (EPG//OPG/LPG) of faeces [21,23]. Briefly, this technique employs the FLOTAC^®^ apparatus and is based on centrifugal flotation of the faecal sample and subsequent translation of the apical portion of the floating suspension. In the FLOTAC^®^ basic technique, both flotation chambers of the FLOTAC^®^ apparatus (10 mL of volume, corresponding to 1 g of feces) are filled with a single flotation solution, in this case FS8 solution [21].

Blood sampling was performed only on birds undergoing other diagnostic tests, for a total of 33 blood samples. On these selected animals, pure alcohol was nebulised over the sampling area, i.e., the right jugular vein, as it is larger than the left, to disinfect the skin and remove feathers for a better visualisation of the blood vessel. With the application of a little pressure at the base of the neck, just above the thoracic inlet, the jugular vein distended, becoming visible through the bird’s thin skin, and the blood was collected using a 2 mL syringe and a 24-gauge needle. When possible, one or more blood smears were immediately prepared on a slide. Then, the collected blood samples were placed in a tube containing the anticoagulant lithium heparin and transported to the laboratory at refrigeration temperature (+4 °C). Once in the laboratory, additional blood smears were prepared from the samples preserved in the anticoagulant, fixed with 100% methanol for 2 min and stained with 5% Giemsa for 45 min for the detection of haemoparasites. Then, the slides were rinsed under cold water, drained, air-dried and observed under a light microscope at 400× and 1000× magnification, under oil immersion, with an average scan time of 10 min for each blood smear examined.

### 2.3. Statistical Analysis

An animal was considered infected if at least one helminth egg or coccidian oocyst was observed. The rates of infected animals and the distributions of eggs and oocysts observed per gram of faeces were calculated by considering the abundance and standard deviation in each sample and for each parasite taxon, except for cestodes [24].

The parasite positivity and the different taxa of detected endoparasites were introduced as dependent variables in generalised linear models (GLMs) with binary logistic response. Data collected in the individual form, including age (young, under one year of age or adult, over one year of age), diet (feeding exclusively with thawed meat or not), family (Accipitridae, Cathartidae, Falconidae, Strigidae or Tytonidae), housing (free in the aviary or tied to a perch), origin of each bird (permanently in captivity or wild animal in temporary captivity for recovery activities) and placement in the aviary (individually, in pairs or in groups) were considered as risk factors. It is worth noting that the age was defined through the observation of behavioural and morphological characteristics, primarily plumage [25].

Firstly, a univariate binary logistic regression analysis was performed to determine factors that could be considered predictors of positivity. In a second step, the variables showing a *p*-value < 0.1 were entered into a multivariate model developed by backward elimination until all remaining variables were significant (*p*-value < 0.05). Similarly, generalised logistic models with binary logistic response for Giemsa-stained blood smear positivity were implemented.

Statistical analysis was carried out using SPSS software (Statistical Package for Social Science, IBM SPSS Statistics for Windows, Version 28.0.1.1, Chicago, IL, USA).

## 3. Results

**Figure 1:**
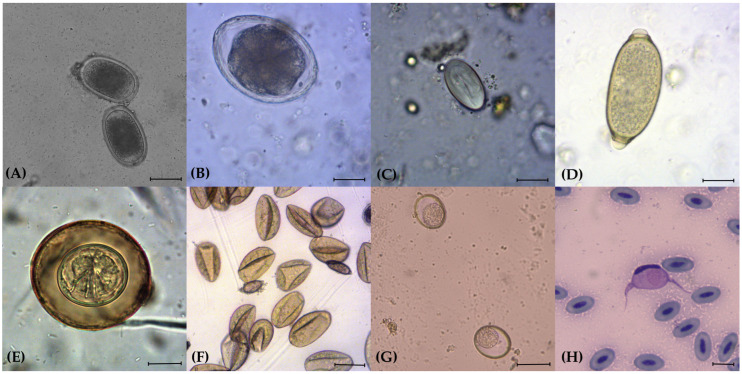
Parasitic elements detected in the analysed faecal and blood samples in captive birds of prey in northern Italy. (**A**) Eggs of Ascarididae (100×); (**B**) egg of *Porrocaecum* spp. (100×); (**C**) egg of Spiruridae (200×); (**D**) egg of Capillariidae (400×); (**E**) egg of Cestoda with visible hooks of the embryo (400×); (**F**) eggs of Trematoda deformed by the contact with the FS8 flotation solution (100×); (**G**) oocysts of *Caryospora* spp. (100×); (**H**) Giemsa-stained blood smear with *Leucocytozoon* spp. (1000×). Scale bars: (**A**–**C**) 20 μm; (**D**–**F**,**H**) 10 μm; (**G**) 40 μm.

Of the 72 faecal samples, 30 (41.7%, 95% CI: 30.1–53.9) were positive for at least one parasite taxon. The prevalences in relation to order of birds of prey were as follows: 50% for Accipitriformes (14/28, 95% CI: 30.6–69.3), 43% for Falconiformes (6/14, 95% CI: 17.7–71.1) and 33.3% for Strigiformes (10/30, 95% CI: 17.3–52.8). As for haemoparasites, an overall prevalence of 21.2% (95% CI: 21.2%, 9–38.9) was recorded, with 7 positive samples out of 33. The main gastrointestinal and blood parasites detected in this study are listed in Table 1 and shown in Figure 1.

Among nematodes, which were the most common helminths found in faecal samples, parasites belonging to the family Strongylidae (19.4%, 95% CI: 11.1–30.5) showed the highest prevalence, followed by those belonging to the families Capillariidae and Spiruridae (both 8.3%, 95% CI: 3.1–17.3). Regarding the family Ascarididae, it was possible to perform a morphological distinction only between eggs of *Heterakis gallinae* and *Ascaridia galli* (referred to as “Other genera”) and those of the genus *Porrocoecum* spp. due to differences in egg size and shape [26].

Coccidia were detected in 12 animals, including four positives for the genus *Caryospora* spp. (5.5%, 95% CI: 2.2–13.4) and eight for the genus *Eimeria* spp. (11.1%, 95% CI: 5.7–20.4); meanwhile, cestodes and trematodes were found in two (2.8%, 95% CI: 0.3–9.7) and four birds (5.6%, 95% CI: 1.5–13.6), respectively. Regarding quantitative results, the examined animals showed a very high excretion of trematode eggs (mean EPG = 967.3); a high individual excretion of Ascarididae and Capillariidae eggs and *Eimeria* spp. oocysts was also observed (Table 2).

According to the order of captive birds of prey, Accipitriformes showed higher positivity to Strongylidae (28.6%, 95% CI: 13.2–48.7), while Falconiformes and Strigiformes had higher prevalences of coccidian oocysts, *Caryospora* spp. (28.6%, 95% CI: 8.4–58.1) and *Eimeria* spp. (20%, 95% CI: 7.7–38.6), respectively (Table S1).

In the univariate analysis, only two of the considered risk factors were statistically significant: age (adult/young) and diet (also fresh meat/only thawed meat) (Table 3), whereas in the multivariate analysis, performed by backward elimination, none of the variables were statistically significant.

As for haemoparasites, *Haemoproteus*/*Plasmodium* spp. infections were identified in seven birds of prey, while *Leucocytozoon* spp. was identified in only one animal, which was also positive for *Haemoproteus*/*Plasmodium* spp. (Table 4). None of the risk factors considered were significant in either the univariate or multivariate analysis.

Considering coinfections, 20 out of 81 birds (24.7%, 95% CI: 15.8–35.5) were infected by more than one type of parasite, including gastrointestinal and blood parasites.

## 4. Discussion

This study documented the first use of the FLOTAC^®^ basic technique on faeces of captive birds of prey, which recorded both the parasite prevalence and the faecal egg count (FEC) in each analysed sample in terms of EPG/OPG. Data obtained by quantifying the parasite loads can be used to carry out epidemiological studies and to target drug treatment in threatened bird populations whose conservation is essential. Indeed, several parasites of birds of prey can be controlled or directly prevented with proper captive management measures, which also include a regular plan of parasitological screening [6,27,28].

Studies on the parasite fauna of birds of prey are scarce and fragmentary, as most of them are protected species for which suitable samples are difficult to obtain [6,19,28]. Regarding the helminths in wild birds of prey, several surveys conducted in different countries reported variable prevalences: between 72.4% and 95% in Italy [8,19], 89.6% in Netherlands [9], 65% in Spain [17], 54.5% in the Slovak Republic [29] and 33.4% in Germany [30]. As for haemoprotozoa, the few studies conducted worldwide recorded prevalences ranging between 11% and 38.5% [14,16,31].

In captive birds of prey, large discrepancies can be observed in the prevalence values of endoparasites, which are usually lower than in wild species (0–13.5%) [7,11,32]. Only two surveys reported higher prevalences, ranging between 46.7% and 100% [10,33]. This variability could be related to different sample sizes, diagnostic methods and flotation solutions. For example, in some investigations, the spontaneous sedimentation and flotation with saturated sodium chloride or nitrate solution (s.g. = 1.200) were used [7,33], while in others, flotation solutions with a higher specific gravity, such as zinc sulphate (s.g. = 1.350), were employed [10].

In our study, 30 positive samples out of 72 (41.7%, 95% CI: 30.1–53.9) were documented by copromicroscopic analysis, demonstrating that parasites can be very common in captive birds of prey. Moreover, the use of FLOTAC^®^ basic technique, employing the FS8 flotation solution (s.g. = 1440), allowed us to detect several parasitic elements, even in cases of low excretion, and to quantify the parasite load.

The prevalence was high on average for all three orders included: 50% for Accipitriformes (14/28, 95% CI: 30.6–69.3), 43% for Falconiformes (6/14, 95% CI: 17.7–71.1) and 33.3% for Strigiformes (10/30, 95% CI: 17.3–52.8). It is important to emphasise that none of the birds examined had clinical symptoms referrable to the presence of parasites.

Among the identified parasites, nematodes were the most frequently recorded, particularly those of the family Strongylidae (19.4, 95% CI: 11.1–30.5), as already highlighted by an Italian survey conducted in central Italy (Table 2) [7]. Strongyles can be found in caeca, gizzard and respiratory tract, and strong infections could lead to serious disease with cachexia, diarrhoea, dyspnoea, head shaking, open mouth breathing and plumage opacity [7,27]. In our survey, they were mainly found in Accipitriformes, while in the other two orders of birds, lower prevalences were recorded (Table S1), although some studies have also identified high prevalences in Falconiformes [34,35]. Eggs of Capillariidae were found in 8.3% (6/72, 95% CI: 3.1–17.3) (Table 2) of the samples and were only detected in Accipitriformes and Strigiformes with prevalence values of 7.1% (95% CI: 0.8–23.5) and 13.3% (95% CI: 3.7–30.7), respectively (Table S1). Species belonging to this family have a wide host range and are characterised by high pathogenicity with clinical signs of anaemia, diarrhoea, regurgitation, weight loss and formation of necrotic plaques along the gastrointestinal tract [27,36]. Spiruridae eggs were detected in 6 out of 72 samples (8.3%, 95% CI: 3.1–17.3) (Table 2); also, these parasites could be responsible for clinical disease, involving several organs, such as the proventriculus and the air sacs [17,19].

As for Ascarididae, the presence of *Porrocaecum* spp. eggs was demonstrated in three animals, while those of other genera were observed in just one animal (Table 2). All positive birds of prey belonged to the orders Accipitriformes and Falconiformes, as shown by another study (Table S1) [30].

The prevalence of cestodes and trematodes was rather low; indeed, only two and four birds of prey were positive, respectively (Table 2): cestodes were found only in Strigiformes, while trematodes were highlighted in Accipitriformes and Falconiformes (Table S1). These parasites are uncommon in captive birds of prey, whereas they are frequently found in free-ranging birds, probably due to an increased exposure to the different intermediate hosts, which are scarcely present in captivity [8,11,31,36].

*Eimeria* spp. oocysts were mainly detected in Strigiformes (20%, 95% CI: 7.7–38.6) (Table S1), as reported in other studies conducted in Mexico and Germany [11,30], while *Caryospora* spp. oocysts were only found in Falconiformes (28.6%, 95% CI: 8.4–58.1), in agreement with data reported in Europe, the Middle East and North America, which demonstrated high prevalences in young captive falcons (Table S1) [30,36]. Unlike other coccidian parasites, *Caryospora* spp. could cause lethargy, diarrhoea, and weight loss in infected animals [36].

The high values of both prevalence and abundance reported in our study could be related to the life in captivity, where bird cages are close to each other and frequently host more than one animal under poor hygienic conditions, enhancing the transmission of infectious diseases. Indeed, many parasites are transmitted via the faecal–oral route and contaminated environment, food and water may play a major role as sources of infection [7,37]. Furthermore, some zoological aviaries usually house birds whose history of disease exposure is often unknown; thus, it is essential to ensure the proper health monitoring of both newly introduced animals and those already present to reduce the spread of new diseases and avoid the risk of multiple infections [11,33,37,38].

According to the univariate model, the statistically significant variables (*p*-value < 0.1) were age and diet (Table 3). As age increases, the risk of finding endoparasites could be higher due to the greater exposure to parasites over time and prolonged life spent in captivity [3]. About diet, it could be hypothesised that birds that are fed only thawed meat would be less susceptible to infection as the cold treatment could favour the reduction of exogenous food contamination.

Regarding blood parasites, 7 out of 33 (21.2%, 95% CI: 9–38.9) birds of prey were positive: one tested positive for both *Leucocytozoon* spp. and *Haemoproteus*/*Plasmodium* spp. and six for *Haemoproteus*/*Plasmodium* spp. (Table 4). Other studies reported comparable values [11,15,39,40]; only a survey conducted in California showed higher prevalences (96%) [41]. A limitation of the study is that *Haemoproteus* and *Plasmodium* spp. were not distinguished by microscopic analysis because some of the slides, despite being examined for several minutes by experienced parasitologists, were of doubtful interpretation. This could be related to the prolonged period of keeping blood in the anticoagulant before smearing.

In general, it is possible to distinguish *Plasmodium* spp. from the genus *Haemoproteus* by the presence of merogony in circulating erythrocytes [42]. Molecular techniques are also useful for detecting blood parasites, particularly in the early stages and during chronic infections when parasitaemia is low, and they may not be visible in blood smears [43].

## 5. Conclusions

In this study, a wide circulation of endoparasites was demonstrated by faecal and blood analysis.

These findings could suggest a high exposure to pathogens in captive birds of prey and the need to further investigate the prevalence and abundance of infections in rehabilitation centres and other facilities. Knowledge of parasites commonly found in birds and the associated risk factors could help develop proper monitoring programs, establish an adequate health assessment plan, including faecal and blood testing, identify current causes of morbidity and mortality and ensure good living and welfare conditions.

## Figures and Tables

**Table 1:** Endoparasites detected by faecal and blood analysis in captive birds of prey in northern Italy, classified by order of belonging (Accipitriformes, Falconiformes and Strigiformes).

Birds of Prey Common Name	Birds of Prey Scientific Name	N° Birds of Prey	N° Faecal Samples	N° Blood Smears	N° Positive Samples/Total	Detected Parasites
**Accipitriformes**						
Cinereous vulture	*Aegypius monachus*	1	1	0	1/1	Spiruridae
Golden eagle	*Aquila chrysaetos*	1	1	0	1/1	*Eimeria* spp.
Bonelli’s eagle	*Aquila fasciata*	1	1	0	0/1	-
Steppe eagle	*Aquila nipalensis*	2	2	0	2/2	*Eimeria* spp., *Porrocaecum* spp., Strongylidae
African hawk-eagle	*Aquila spilogaster*	1	0	1	0/1	-
Eurasian buzzard	*Buteo buteo*	3	3	3	2/3	Ascarididae, Capillariidae, *Porrocaecum* spp., *Haemoproteus*/*Plasmodium* spp., *Leucocytozoon* spp., Trematoda
Red-tailed hawk	*Buteo jamaicensis*	2	2	1	1/2	Strongylidae
Ferruginous hawk	*Buteo regalis*	1	0	1	0/1	-
Turkey vulture	*Cathartes aura*	1	1	0	1/1	Spiruridae
Black-chested buzzard-eagle	*Geranoaetus* *melanoleucus*	1	1	0	1/1	Strongylidae
White-backed vulture	*Gyps africanus*	1	1	0	0/1	-
Bald eagle	*Haliaeetus* *leucocephalus*	3	3	1	2/3	Strongylidae, Trematoda
Harris’s hawk	*Parabuteo unicinctus*	11	11	6	3/11	Strongylidae
White-headed vulture	*Trigonoceps occipitalis*	1	1	0	0/1	-
**Falconiformes**						
Crested caracara	*Caracara cheriway*	3	2	1	0/3	-
Lanner falcon	*Falco biarmicus*	2	2	0	0/2	-
Barbary falcon	*Falco pelegrinoides*	1	1	0	0/1	-
Peregrine falcon	*Falco peregrinus*	1	1	0	0/1	-
Eurasian hobby	*Falco subbuteo*	2	2	2	2/2	*Haemoproteus*/*Plasmodium* spp., Spiruridae
Common kestrel	*Falco tinnunculus*	2	1	2	2/2	Spiruridae, Strongylidae, Trematoda
Gyrfalcon/saker falcon (hybrid)	*Falco rusticolus/Falco cherrug*	3	3	0	3/3	*Caryospora* spp.
Gyrfalcon/lanner falcon (hybrid)	*Falco rusticolus/Falco biarmicus*	3	2	2	1/3	*Caryospora* spp., *Porrocaecum* spp.
**Strigiformes**						
Northern long-eared owl	*Asio otus*	1	1	1	0/1	-
Little owl	*Athene noctua*	8	8	6	2/8	*Haemoproteus*/*Plasmodium* spp.
Rock eagle-owl	*Bubo bengalensis*	1	1	1	1/1	*Eimeria* spp.
Eurasian eagle-owl	*Bubo bubo*	12	8	1	2/12	*Eimeria* spp., Strongylidae
Snowy owl	*Bubo scandiacus*	4	4	0	3/4	Capillariidae, *Eimeria* spp., Spiruridae, Strongylidae
Great horned owl	*Bubo virginianus*	1	1	0	1/1	Capillariidae, Cestoda, *Eimeria* spp., Strongylidae
Great horned owl subarcticus	*Bubo virginianus subarcticus*	1	1	0	1/1	Capillariidae, *Eimeria* spp., Strongylidae
Eurasian scops owl	*Otus scops*	2	2	1	2/2	Capillariidae, Cestoda
Common barn owl	*Tyto alba*	4	4	3	0/4	-

**Table 2:** Prevalences and abundances of endoparasites detected by faecal analysis in captive birds of prey in northern Italy.

**Phylum**	**Family**	**Genus**	**N° Positive** **Samples/Total**	**Prevalence (95% CI** ^**a**^**)**	**EPG/OPG** ^**b**^ **(SD** ^**c**^**)**	**Min–Max**
Nematoda	Ascarididae	*Porrocaecum* spp.	4/72	5.6 (1.5–13.6)	39.7 (281.9)	0–2368
		Other genera	1/72	1.4 (0.03–7.5)	84.2 (714.6)	0–6064
	Capillariidae	Nd ^d^	6/72	8.3 (3.1–17.3)	86.6 (519.3)	0–3888
	Spiruridae	Nd	6/72	8.3 (3.1–17.3)	9.6 (58.7)	0–440
	Strongylidae	Nd	14/72	19.4 (11.1–30.5)	15.63 (71.1)	0–512
**Phylum**	**Class**		**N° Positive** **Samples/Total**	**Prevalence (95% CI** ^**a**^**)**	**EPG/OPG** ^**b**^ **(SD** ^**c**^**)**	**Min–Max**
Platyhelminthes	Cestoda		2/72	2.8 (0.3–9.7)	Nd	Nd
	Trematoda		4/72	5.6 (1.5–13.6)	967.3 (796.5)	0–67,584
**Phylum**	**Subclass**	**Genus**	**N° Positive** **Samples/Total**	**Prevalence (95% CI** ^**a**^**)**	**EPG/OPG** ^**b**^ **(SD** ^**c**^**)**	**Min–Max**
Apicomplexa	Coccidia	*Caryspora* spp.	4/72	5.5 (2.2–13.4)	48.9 (403.5)	0–3424
		*Eimeria* spp.	8/72	11.1 (5.7–20.4)	247.6 (2100.9)	0–17,952
Total			30/72	41.7 (30.1–53.9)		

^a^ CI: Confidence interval; ^b^ EPG/OPG: eggs per gram/oocysts per gram; ^c^ SD: standard deviation; ^d^ Nd: not determined.

**Table 3:** Potential risk factors associated with parasite prevalence by a univariate analysis in captive birds of prey in northern Italy. Test performed at a 95% significance level; *p*-value ≤ 0.1 was considered significant.

Risk Factor	Category	*p*-Value	OR ^a^	95% CI ^b^
Age	Adult	<0.05 *	15.20	1.54–150.4
	Young	1		
Diet	Also fresh meat	<0.05 *	35.4	1.6–771.2
	Only thawed meat	1		
Family	Accipitridae	>0.05	1.4 × 10^10^	∞
	Cathartidae	>0.05	1.1 × 10^21^	∞
	Falconidae	>0.05	3.7 × 10^9^	∞
	Strigidae	>0.05	1 × 10^10^	0
	Tytonidae	1		
Housing	Free in the aviary	>0.05	11	0.54–226.3
	Tied to a perch	1		
Origin	Permanently in captivity	>0.05	7.2	0.35–150.4
	Wild temporarily in captivity	1		
Placement in aviary	In groups	>0.05	2.21	0.23–20.9
	In pairs	>0.05	0.075	0.002–2.7
	Individually	1		

^a^ OR: Odds ratio; ^b^ CI: confidence interval; * significant value.

**Table 4:** Haemoparasites detected in blood smears in captive birds of prey in northern Italy.

Order of Birds of Prey	N° Positive Samples/Total (Prevalence, 95% CI ^a^)	Species of Bird of Prey	*Haemoproteus*/*Plasmodium* spp.	*Leucocytozoon* spp.
Accipitriformes	2/13	*Buteo buteo*	2	1
Falconiformes	4/7	*Falco subbuteo*	2	0
		*Falco tinnunculus*	2	0
Strigiformes	1/13	*Athene noctua*	1	0
Total	7/33 (21.2%, 9–38.9)		7/33	1/33

^a^ CI: Confidence interval.

## Data Availability

The original contributions presented in the study are included in the article/supplementary material, further inquiries can be directed to the corresponding author.

## Supplementary Materials

The following supporting information can be downloaded at: https://www.mdpi.com/article/10.3390/ani14243579/s1, Table S1: Prevalences of parasites detected by faecal analysis in captive birds of prey in northern Italy according to order of belonging (Accipitriformes, Falconiformes and Strigiformes).
