# Supplementary material for: Investigating Endoparasites in Captive Birds of Prey in Italy
Source: Animals (Basel). 2024 Dec 11;14(24):3579. doi: 10.3390/ani14243579 (PMC11672671; doi:10.3390/ani14243579)
Supplement: Supplementary file 1 [file animals-14-03579-s001.zip › animals-3324679-supplementary.pdf]

Table S1. Prevalences of parasites detected by faecal analysis in captive birds of prey in northern Italy according to order of belonging (Accipitriformes, Falconiformes and Strigiformes).

Order of Birds of Prey	Detected Parasites		N° Positive Samples/Total	Prevalence (95% CI [a])
Accipitriformes	Strongylidae		8/72	11.1% (5.7–20.4)
	Capillariidae		2/72	2.8% (0.3–9.7)
	Ascarididae	*Porrocaecum* spp.	3/72	4.2% (1.4–11.5)
		Other genera	1/72	1.4 (0.03–7.5)
	Spiruridae		3/72	4.2% (1.4–11.5)
	Cestoda		0/72	0
	Trematoda		3/72	4.2% (1.4–11.5)
	Coccidia	*Caryospora* spp.	0/72	0
		Eimeria spp.	2/72	2.8% (0.3–9.7)
Falconiformes	Strongylidae		1/72	1.4 (0.03–7.5)
	Capillariidae		0/72	0
	Ascarididae	*Porrocaecum* spp.	1/72	1.4 (0.03–7.5)
		Other genera	0/72	0
	Spiruridae		2/72	2.8% (0.3–9.7)
	Cestoda		0/72	0
	Trematoda		1/72	1.4 (0.03–7.5)
	Coccidia	*Caryospora* spp.	4/72	5.6% (1.5–13.6)
		Eimeria spp.	0/72	0
Strigiformes	Strongylidae		5/72	6.9% (3–15.2)
	Capillariidae		4/72	5.6% (1.5–13.6)
	Ascarididae	*Porrocaecum* spp.	0/72	0
		Other genera	0/72	0
	Spiruridae		1/72	1.4 (0.03–7.5)
	Cestoda		2/72	2.8% (0.3–9.7)
	Trematoda		0/72	0
	Coccidia	*Caryospora* spp.	0/72	0
		Eimeria spp.	6/72	8.3% (3.1–17.3)

[a] CI: Confidence Interval
